# *Mycobacterium abscessus*, an Emerging and Worrisome Pathogen among Cystic Fibrosis Patients

**DOI:** 10.3390/ijms20235868

**Published:** 2019-11-22

**Authors:** Giulia Degiacomi, José Camilla Sammartino, Laurent Roberto Chiarelli, Olga Riabova, Vadim Makarov, Maria Rosalia Pasca

**Affiliations:** 1 Department of Biology and Biotechnology “Lazzaro Spallanzani”, University of Pavia, 27100 Pavia, Italy; giulia.degiacomi@unipv.it (G.D.); jose.sammartino@iusspavia.it (J.C.S.); laurent.chiarelli@unipv.it (L.R.C.); 2 IUSS—University School for Advanced Studies, 27100 Pavia, Italy; 3 Bach Institute of Biochemistry, Research Center of Biotechnology of the Russian Academy of Sciences, 119071 Moscow, Russia; obr1973@mail.ru (O.R.); makar-cl@ropnet.ru (V.M.)

**Keywords:** *Mycobacterium abscessus*, cystic fibrosis, drug resistance, nontuberculous mycobacteria

## Abstract

Nontuberculous mycobacteria (NTM) have recently emerged as important pathogens among cystic fibrosis (CF) patients worldwide. *Mycobacterium abscessus* is becoming the most worrisome NTM in this cohort of patients and recent findings clarified why this pathogen is so prone to this disease. *M. abscessus* drug therapy takes up to 2 years and its failure causes an accelerated lung function decline. The *M. abscessus* colonization of lung alveoli begins with smooth strains producing glycopeptidolipids and biofilm, whilst in the invasive infection, “rough” mutants are responsible for the production of trehalose dimycolate, and consequently, cording formation. Human-to-human *M. abscessus* transmission was demonstrated among geographically separated CF patients by whole-genome sequencing of clinical isolates worldwide. Using a *M. abscessus* infected CF zebrafish model, it was demonstrated that *CFTR* (cystic fibrosis transmembrane conductance regulator) dysfunction seems to have a specific role in the immune control of *M. abscessus* infections only. This pathogen is also intrinsically resistant to many drugs, thanks to its physiology and to the acquisition of new mechanisms of drug resistance. Few new compounds or drug formulations active against *M. abscessus* are present in preclinical and clinical development, but recently alternative strategies have been investigated, such as phage therapy and the use of β-lactamase inhibitors.

## 1. Introduction

Cystic fibrosis (CF) is an autosomal recessive disease characterized by the involvement of respiratory, gastrointestinal, and male reproductive tracts, even if most of the morbidity and mortality arises from CF lung disease [1,2]. In particular, thick airway secretions impair the mucociliary clearance, which increases bacterial colonization and infection [3]. 

In this context, nontuberculous mycobacteria (NTM) have recently emerged as important pathogens in CF lung disease worldwide [2,3,4]. Over the last two decades, the incidence of NTM infections among CF patients has raised from 3.3% to 22.6%, increasing morbidity and mortality associated with these pathogens [4,5,6]. At the same time, for other CF pathogens, such as *Pseudomonas aeruginosa* and *Burkholderia cepacia*, the incidence has significantly decreased [7,8]. Additionally, the NTM incidence is underestimated because of misdiagnosis of NTM infections such as tuberculosis (TB) is common in developing countries; moreover, data from several countries are missing [7,8]. 

The most commonly identified NTM species in CF individuals are the slow growing *Mycobacterium avium* complex (MAC) and the rapidly growing *Mycobacterium abscessus* complex (MABSC) (95% of CF cases) [2,3,4]. MABSC is more common in European CF populations and its incidence is globally increasing; moreover, it is frequently found in younger CF patients (including children) and in those with more severe lung disease [4,9,10]. MABSC includes the following *M. abscessus* subspecies: *Mycobacterium abscessus* subsp. *abscessus* (*Mab*), *Mycobacterium abscessus* subsp. *bolletii* (*M. bolletii*), and *Mycobacterium abscessus* subsp. *massiliense* (*M. massiliense*). The MAC predominantly consists of *Mycobacterium avium* subsp. *avium* (*Mav*) and *Mycobacterium avium* subsp. *intracellulare* [2,3,4,5,6]. 

Among NTM subspecies, *Mab* is becoming the most prominent and worrisome pathogen in hospitals and CF centers around the world [4,7,10,11]. It is the major NTM causing respiratory infections worldwide (up to 80%), most often in immunocompromised patients, such as those with CF and HIV-positive status, and in patients with chronic obstructive pulmonary disease (COPD) and bronchiectasis [4,7,10,11,12,13,14,15,16]. 

*Mab* drug therapy takes up to 2 years (see below), with only about 30% of patients experiencing successful treatment outcomes [11,17]. *Mab* treatment is also challenging, since failed eradication leads to an accelerated lung function decline. *Mab*-infected CF patients are even excluded from lung transplant lists in some countries. Consequently, in this cohort of patients, preventing *Mab* infection is essential. Room cleaning protocols have recently been changed and *Mab*-infected CF patients are now hospitalized in specialized CF centers, where there are 15 independent air changes per hour in the rooms to remove potentially infectious aerosol, thus preventing transmission [12].

**Figure 1:**
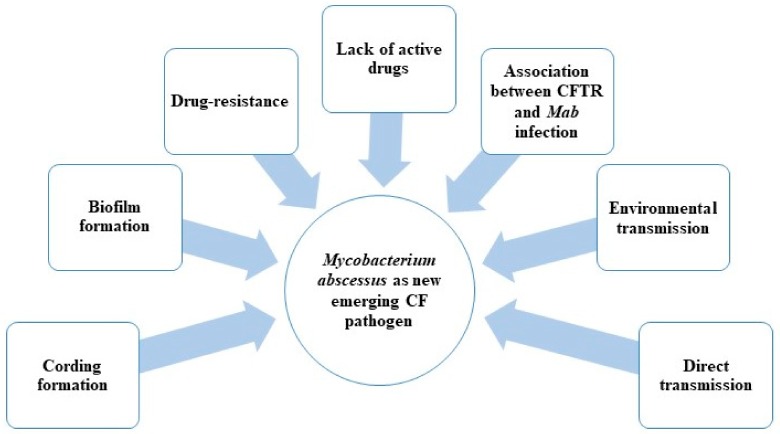
Factors contributing to the spread of *M. abscessus* (*Mab*) as an emerging pathogen among cystic fibrosis (CF) patients. CFTR: cystic fibrosis transmembrane conductance regulator.

*Mab*’s success in becoming an emerging CF pathogen is due to several reasons, including:

1. Possible direct person-to-person transmission;
2. Biofilm and drug resistance;
3. Association between *CFTR* (cystic fibrosis transmembrane conductance regulator) mutations and formation of granuloma in the presence of *Mab* infection;
4. Lack of active drugs (in particular with bactericidal activity) (Figure 1).

In this review, we will focus on analyzing all of these aspects in order to find a possible “Achilles’ heel” to fight this emerging pathogen.

## 2. Possible *M. abscessus* Direct Transmission among CF Patients

*Mab*, similar to other NTM subspecies, is ubiquitous in the environment, such as in soil and drinking water, and remains viable even after water treatment. Therefore, this pathogen can survive in environments near to human populations, particularly in human water sources, including hospital and domestic water supplies [16]. *Mab* and other NTM subspecies are also commonly found in urban water plumbing and water systems, sometimes in symbiosis with Amoebae [18,19,20,21,22,23,24,25,26,27]. Moreover, *Mab* has been isolated from fish [28,29,30,31,32,33] and animals [34,35,36,37,38,39,40], who could also represent reservoirs for human infections. This makes exposure common and disinfection difficult, which is very problematic in healthcare settings [7,15,16,19,23].

However, in sporadic and epidemic *Mab* infections, the pathogen is almost never isolated from the closest environment [7]. 

Until recently, it was believed that among CF patients, the majority of *Mab* infections were acquired by individuals through exposure to soil, household dust, or water, potentially via fomites and aerosols [41]. The mode of *Mab* transmission is still under investigation, and only recently was human-to-human *Mab* transmission demonstrated using whole-genome sequencing (WGS) [11,14,42].

In fact, Bryant et al. (2016) [14], using WGS of prominent worldwide *Mab* clinical isolates, showed that the majority of infections were acquired through direct transmission, potentially via fomites and aerosols. In particular, they generated WGS of 1080 *Mab* clinical isolates from 517 patients, obtained from CF centers from Europe, the United States, and Australia. They also identified that 74% of isolates were clustered in three dominant circulating clones: *Mab* clusters 1 and 2 and *M. massiliense* cluster 1. These 3 clusters were present in all CF centers, indicating transcontinental spreading of these strains by a possible human-to-human transmission within the global CF patient community. The clustered strains presented less than 20 single-nucleotide polymorphisms (SNPs), indicating a high level of human-to-human transmission among geographically separated CF patients [14]. Interestingly, these clustered *Mab* isolates were associated with bad clinical outcomes and presented increased virulence in vivo, thus representing an urgent international challenge [14]. 

According to the previous study, Yan et al. (2019) performed WGS of *Mab* isolates from 22 CF patients [43]. WGS identified a cluster of three CF patients infected by *Mab* isolates that differed by < 7 SNPs, suggesting a possible direct transmission among them. Several hospital attendances were found in common for these 3 patients, even if they were hospitalized in separate single rooms and there were no known social links between them [43]. The genomes of these *Mab* isolates are very similar to those previously described, confirming the presence of global circulating *Mab* clones in CF centers [14].

An additional study evaluated the transmission of *Mab* isolates in 4 Italian CF centers using the WGS of clinical isolates [44]. They found 7 possible person-to-person transmissions (SNP difference cut-off of < 30); only three CF patients were hospitalized in the same CF center at the same time [44]. Moreover, one of the *Mab* clusters identified in this study is the same as cluster 1 detected by Bryant and collaborators [14,44], again highlighting the presence of circulating virulent *Mab* strains worldwide in CF centers.

These last studies [14,43,44] show how it is possible to monitor human-to-human *Mab* transmission by WGS approach only, and to ascertain if different patients, even if geographically separated, share the same strain. *Mab* and *M. massiliense* belong to the same complex (MABSC) [2]; at this point, it is important to underline that the possibility of direct transmission for *M. massiliense* subspecies was also shown by genomic approach [14]. How human-to-human direct *Mab* transmission occurs is still under investigation.

Moreover, the human-to-human transmission helped *Mab* evolution from an environmental bacterium to a transmissible human pathogen. This evolution also affected the strategies used in CF centers to contain *Mab* infections. Before this last discovery, effective sterilizing techniques and other hygiene practices were performed to reduce the risk of environmental *Mab* transmission. Now, the *Mab* infection control recommendations include general infection control measures (e.g., hand washing) and advise the segregation of infected CF patients from the other ones in order to avoid direct transmission [12].

## 3. Pathogenesis of *M. abscessus*

The most common *Mab* infection sites are the respiratory tract, the skin, and soft tissue [4]. 

It is well known that predisposing factors for *Mab* pulmonary infections are chronic bronchiectasis and CF disease, as in these conditions the pathogen can firstly develop a biofilm, colonizing the host, and later progress into an invasive disease [45,46,47]. The progress of *Mab* infection in the CF lung is still under investigation [47], but a recent study showed that *Mab* aggregates form a biofilm around lung alveoli [48]. It is noteworthy that mycobacteria growing in biofilms are more tolerant to antibiotics, contributing to their drug resistance [49].

Moreover, the *Mab* cell wall contributes largely to its drug resistance and to its pathogenicity, thanks to the large presence of complex lipids, among which are five major glycopeptidolipids (GPLs) that differ in location or number of acetyl and sugar moieties [49,50,51,52,53]. 

Because of its peculiar cell wall, *Mab* shows smooth (S) or rough (R) colony morphologies, associated with distinct in vitro and in vivo features. In particular, the wild-type S strains produce abundant GPLs and minimal trehalose dimycolate (TDM), whilst R mutants have genetic lesions in the GPL loci, producing little or no GPLs and higher levels of TDM [49,50,53,54]. TDM, contributing also to the *Mycobacterium tuberculosis* virulence, was found on the surface of *Mab* R strains [53,54]. In fact, TDM is responsible for the cording phenotype, a key factor in increasing *Mab* virulence, causing invasive infection [53]. The lack of cording in the S variant may be due to the presence of GLPs masking other cell surface molecules (e.g., TDM) that activate innate immunity, causing inhibition of the macrophage apoptotic response, reduced production of radical oxygen species (ROS), and limitation of the spread of *Mab* among macrophages [49,50,51,52,53,54,55]. Furthermore, the high presence of GLPs in S strains is responsible for the formation of a robust biofilm during the infection [48,49]. 

The *gpl* locus is highly conserved in *Mycobacterium smegmatis*, *Mab*, and *Mav* [50]. This locus contains *mmpS4*, *mmpL4a*, and *mmpL4b* genes, which encode membrane proteins essential for the GPL biosynthesis and transport across the plasma membrane [50,56,57]. The transition from high-GPL (S strains) to low-GPL producers (R strains) is linked to mutations in genes involved in GPL biosynthesis and transport, similar to in the crucial Tyr/Asp couples in MmpL4a/MmpL4b. In fact, it was shown that point mutations in MmpL4a at Tyr842 or MmpL4b at Tyr854 caused loss of GPL production, suggesting that no functional redundancy exists between MmpL4a and MmpL4b [56]; moreover, the disruption of *mmpL4b* in *Mab* S strain inhibits GPL production, causing an R morphotype [58]. Then, the *Mab* S-to-R transition leads to the following events:

- Arrest of lipid transport;
- Production of serpentine cords;
- Growth as extracellular cords, allowing escape from the innate immune defenses;
- Induction of a strong humoral response that contributes to acute and severe infections [51,53,54,55,58].

The high virulence of *Mab* R mutants was confirmed in in vivo infection models, such as with zebrafish, where the transition to the R morphotype is characterized by an increased virulence because of the cording formation, which is responsible for invasive infection and larval death [55,56,59,60]. Moreover, it was found in a zebrafish model that the inactivation of *MAB_4780*, encoding a dehydratase required for cording formation, strongly affects *Mab* R pathogenicity; this attenuation causes both cord deficiency and intracellular growth impairment [60]. Furthermore, the *Mab* R strains induce more aggressive and invasive pulmonary disease, particularly in CF patients; in fact, these mutants are more frequently isolated after a long persistent infection and are associated with increased lung function decline [48,53,61,62,63,64]. 

Consequently, the possibility of transition between S and R morphotypes is very relevant in *Mab* pulmonary infection in CF patients [59,61,62]. These patients are particularly susceptible to colonization by biofilm-forming bacteria because of their altered lung physiology. In these conditions, *Mab* S strains expressing GPL may be favored [65]. A rough cord-forming variant could emerge from CF patients chronically colonized with an S strain, giving a more aggressive, invasive pulmonary infection [60,61,62,63].

The presence of the two morphotypes in *Mab* is also fundamental to its escape from the innate immune system. In particular, the innate immune response to pulmonary pathogens is mediated by the expression of TLR2 and then of IL-8 and human b-defensin 2 (HbD2) from the respiratory epithelial cells. It was shown that only *Mab* R strain stimulates the expression of IL-8 and HbD2, while *Mab* S variant is able to “mask’’ the bioactive cell wall lipids with GPLs. Because S variants are predominant during the first phases of infection, *Mab* is then able to avoid the innate immune system [51].

Another *Mab* peculiarity is that although it is a rapid grower, it persists in lungs associated with granulomatous lesions, a landmark of *M. tuberculosis* infection. Recently, Dubois et al. (2018) showed that an *mmpL8* deletion mutant presented a decreased intracellular viability in a zebrafish model and a diminished propensity to induce granuloma formation [66]; moreover, this mutant had impaired adhesion to macrophages. In fact, MmpL8 is also required for the production of a glycolipid (glycosyl diacylated nonadecyl diol alcohol (GDND)), which is derived from a combination of oleic and stearic acids. In *mmpL8* knock-out (KO) mutant, the reduced GDND production could be the cause of the modified interaction between bacteria and macrophages, resulting in a decreased virulence [66]. 

Finally, Laencina and collaborators (2018) [67] demonstrated that genes belonging to *Mab* ESX-4 locus are essential for its intracellular survival inside amoebae and macrophages. Interestingly, a *Mab* mutant with a deletion in *eccB4* gene, coding for a structural key ESX component, was attenuated. In fact, it was less efficient at blocking phagosomal acidification and failed to damage phagosomes. The authors speculated that because *Mab* lacks ESX-1, ESX-4 could be a surrogate of *M. tuberculosis* ESX-1 [67]. 

### CFTR Mutations Specialize M. abscessus as CF Pathogen

Recently, steps were taken to decipher why CF patients are so predisposed to *Mab* infections. Using zebrafish model, Bernut and collaborators (2016) showed that both macrophages and neutrophils are required to control *Mab* infection; moreover, impaired TNF signaling produced aberrant granulomas, and subsequent larval death [68]. 

In 2019, the same research group demonstrated that *CFTR* participates in neutrophil chemotaxis to the infected *Mab* sites, stimulating the oxidative host defenses [69]. In fact, in a zebrafish model, *Mab* infection was characterized by the recruitment of the bacilli by macrophages; in particular, the activation of macrophages resulted in neutrophil chemotaxis leading to granuloma formation, and ROS production by NOX2, which led to intracellular *Mab* death. In these conditions, the granuloma sequestered *Mab*, containing the infection [69]. Otherwise, *Mab*-infected, *CFTR*-depleted zebrafish were rapidly infected; in this case, the *CFTR* dysfunction reduced both macrophage bactericidal activity and neutrophil recruitment to form the protective granulomas [69]. Interestingly, these findings are only observed with *Mab* and not with other mycobacteria; consequently, *CFTR* seems to have a specific role in the immune control of only *Mab* infections [68,69]. 

These findings clarified the increasing emergence of *Mab* as a CF pathogen. Therefore, *Mab*’s intrinsic drug resistance often results in long therapies and poor clinical outcomes in CF patients [17,70,71], accelerating lung function decline at a greater rate than other bacteria, including *Pseudomonas aeruginosa* and *Burkholderia cepacia* [8,63,65]. 

*Mab* is often called the “incurable nightmare” because the cure rate among CF patients with *Mab* pulmonary infection is only 25%–58% [8,64,65]. CF patients with *Mab* infection are more likely to require transplant or to die despite adequate treatment [65,71,72]. However, CF patients with pre-transplant *Mab* infection could develop post-transplant invasive *Mab* disease. In selected CF patients, surgical resection of infected lung tissue could be beneficial. Consequently, progressive *Mab* disease, despite antibiotic therapy, is considered as a contraindication for lung transplantation by several CF centers, and is associated with treatment failure and increased mortality [65,70,71,72]. 

Recently, the U.S. Food and Drug Administration (FDA) approved Trikafta (elexacaftor/tezacaftor/ivacaftor) for the treatment of CF in people aged 12 years and older who have at least one F508del mutation in the *CFTR* gene. The approval of Trikafta was supported by positive results of two global Phase 3 studies [73,74]. 

If the association among *CFTR* mutation and *Mab* infection is validated, indirectly, Trikafta could also protect CF patients from this pathogen. For this reason, it is mandatory to investigate this topic further, in particular for CF patients.

## 4. Current Therapy against *M. abscessus* Infections

Unfortunately, antitubercular drug use is limited in the management of *Mab* infections, since this bacterium possesses extremely high intrinsic and acquired antibiotic resistance, making its eradication more difficult. *Mab* drug treatment in CF is even more challenging because thick mucus secretions cause an increased renal drug clearance and a possible decreased gastrointestinal absorption [17,71]. 

**Figure 2:**
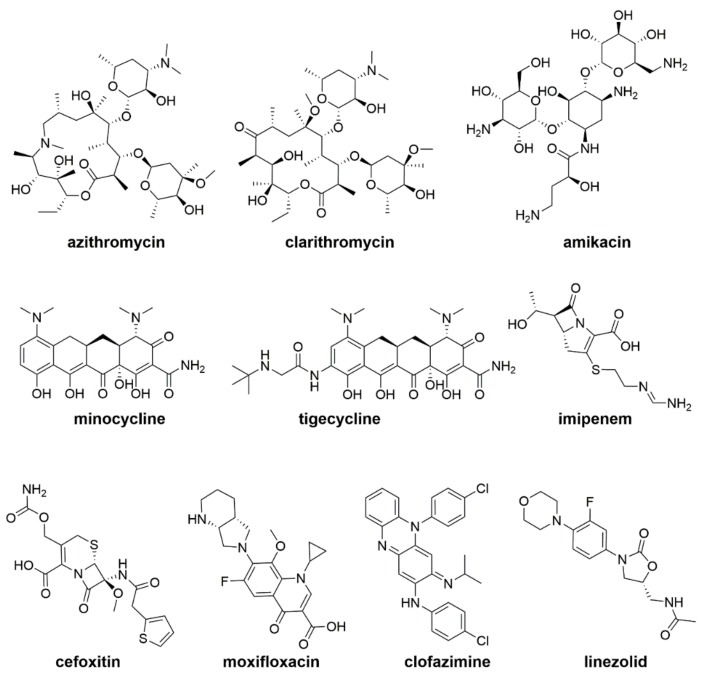
Drugs currently used in *Mab* therapy.

In 2016, the recommendations for NTM management in CF were published [17,71]. The treatment duration was 1 year following culture conversion (the time of conversion starts from the date of the first of three consecutive negative cultures) [17,71]. *Mab* treatment consists of an intensive phase of therapy followed by a continuation phase. The intensive phase should include daily oral macrolide treatment (preferably azithromycin) in conjunction with 3–12 weeks of intravenous (IV) amikacin and one or more of the following antibiotics: IV tigecycline, imipenem, or cefoxitin. The duration of the intensive phase of therapy depends on the type of infection and the tolerability of the treatment [71]. The continuation phase includes the following drugs: inhaled amikacin and a quotidian oral macrolide (preferably azithromycin), in addition to 2–3 oral antibiotics (to be chosen from minocycline, clofazimine, moxifloxacin, and linezolid) [71] (Figure 2). 

Moreover, in agreement with the current American Thoracic Society guidelines, in order to prevent the emergence of macrolide resistance, clarithromycin and azithromycin must be prescribed in combination with other drugs [17,71]. Lastly, the interactions with other chronic medications could affect the tolerance and efficacy of the antibiotic therapy [2].

### Mechanisms of Resistance to Drugs Used in Therapy

As mentioned above, *Mab* is intrinsically resistant to many drugs, including several antitubercular drugs, because of its physiology. At the same time, this pathogen acquired new mechanisms of drug resistance through genomic mutations. In fact, the long drug treatment contributed to the spreading of drug-resistant strains caused by development of mutations either in the target or in other related genes [8,11,75,76]. In this way, drug efficacy is seriously compromised. Several factors contribute to its intrinsic and acquired drug resistance, such as target gene mutations, drug efflux, an impermeable cell wall, and antibiotic-modifying or -inactivating enzymes [11,75,76]. Moreover, particularly among CF patients, respiratory habitats where *Mab* is very close to other pathogens (for example, *P. aeruginosa*) could represent reservoirs for transfer of novel drug resistance or virulence genes [75].

The most important and common mechanisms of acquired resistance to drugs used in *Mab* therapy are reported below (Table 1).

The macrolides, which are used in *Mab* therapy, bind in the peptide exit tunnel of the ribosome, preventing the growth of the peptide chain and consequently inhibiting protein synthesis [88]. In fact, in several *Mab* isolates, high levels of macrolide resistance are linked to mutations in the peptidyltransferase-binding region of the 23S rRNA gene [77,88]. Critical mutations in nucleotides 2058 and 2059, which are involved in the binding of macrolides to ribosomes, are a frequent cause of constitutive macrolide resistance in *Mab* [78]. In fact, the acquisition of 23S rRNA gene mutations during therapy with macrolides has been reported for several NTM species, in particular for *Mab* [77,89].

*Mab erm(41)* gene encodes a methyl-transferase that modifies the clarithromicin ribosomal binding site, causing resistance [77]. In particular, the inactivating enzyme Erm(41) methylates A2058 in the peptidyltransferase region of the 23S rRNA (the drug target), preventing the binding with macrolides [8,77,88]. The T28C polymorphism in *erm(41)* sequence is responsible for the inducible macrolide resistance in *Mab* [78,79]. The long duration of *Mab* macrolide therapy could be the major cause of the spreading of constitutively macrolide-resistant isolates [77,78,88,89]. This mechanism of drug resistance is not present in *M. massiliense*, since it harbors a deletion in the *erm(41)* gene [77,89].

The mechanism of resistance to aminoglycosides in *Mab* is mainly based on the modification of the 30S subunit of the ribosome (the drug target); in fact, the 16S rRNA (*rrs*) and *rpsL* genes are mutated in the 90% of cases of aminoglycoside resistance. In particular, mutations at position 1408 of the *rrs* gene in *Mab* clinical isolates are associated with aminoglycoside resistance [8,80]. In addition, other *Mab* mutations related to high level of aminoglycoside resistance were isolated *in vitro* in the *rrs* gene at position 1406, 1409, and 1491 [81].

Furthermore, the enzymatic drug modification could be the cause of *Mab* aminoglycoside resistance. The *Mab* 20-Nacetyltransferase (AAC(2′)), encoded by *MAB_4395* gene, is able to acetylate several aminoglycosides. In fact, a *Mab* strain harboring a deletion of *MAB_4395* is more sensitive to aminoglycosides (4–64 fold reduction in the Minimum Inhibitory Concentration (MIC)) [75]. Moreover, another N-acetyltransferase, named Eis2 (coded by *MAB_4532c*), is able to modify aminoglycosides, conferring resistance in *Mab* strains [75].

Cefoxitin (cephalosporin) and imipenem (carbapenem) are the only two β-lactams used in therapy against *Mab*. Regrettably, this pathogen produces a strong, constitutive class A β-lactamase (Bla_Mab, encoded by *MAB_2875*) responsible for β-lactam resistance [82]. Imipenem and cefoxitin are hydrolyzed at a very slow rate by Bla_Mab, contributing to their clinical activity. 

Tetracycline affects bacterial protein synthesis by binding the 30S ribosomal subunit and interfering with the delivery of aminoacylated tRNA to the A-site. *Mab* resistance to tetracycline is conferred by the tetracycline inactivating monooxygenase MabTetX (coded by *MAB_1496c*) [83]. Sublethal concentrations of tetracycline confer a strong induction of MabTetX; a strain with a deletion in *MAB_1496c* is highly sensitive to this drug. However, tigecycline, a glycylcycline tetracycline, is a poor substrate of MabTetX and is not able to induce the expression of MabTetX [83].

Clofazimine and bedaquiline are two drugs used for TB treatment; recently, clofazimine was introduced in *Mab* therapy, whilst bedaquiline is under preclinical evaluation. Both drugs have a common mechanism of resistance in both *M. tuberculosis* and *Mab* [84,90], consisting of mutations in the gene coding for the repressor of the efflux pump MmpS5-MmpL5 (*Rv0678* in *M. tuberculosis* and *MAB_2299c* in *Mab*) [84,90]. 

Mutations in *gyrA*, which codes for the fluoroquinolone target DNA gyrase, have been related to resistance in *Mab* [85,86]. However, in a recent study, in which 105 MAC or MABC clinical isolates were analyzed, including 72 resistant to moxifloxacin, no clear correlation was found between mutations in *gyrA* and *gyrB* genes and fluoroquinolone resistance, indicating that other new mechanisms of resistance should be involved [86].

Linezolid, belonging to the oxazolidinone class, is active against *Mab* growth. The main mechanism of resistance involves mutations in 23S rRNA (coded by *rrl*- *MAB_r5052*), the drug target, and in ribosomal proteins (L3, L4, and L22) [87]. Currently, the contribution of some efflux pumps to linezolid resistance is being also investigated [87].

The WhiB7 regulator, encoded by *MAB_3508c*, is a multi-drug inducible transcriptional regulator that activates the expression of genes, conferring aminoglycoside and macrolide resistance (*eis2* and *erm(41)*, respectively) in *Mab* [8,79]. WhiB7 is strongly induced when exposed to antibiotics that target the ribosome, such as erythromycin, clarithromycin, amikacin, tetracycline, and spectinomycin. The strong induction of WhiB7 confers amikacin and clarithromycin resistance; deletion of *MAB_3508c* gene renders *Mab* more susceptible to amikacin and clarithromycin [8,79].

## 5. New Drugs and New Treatments in Preclinical and Clinical Trials

As reported above, the acquired mechanisms of resistance to drugs used in therapy limit the efficacy of the current *Mab* treatment; for this reason, new active compounds with a novel mechanism of action are needed.

Choo et al. (2014) [91] demonstrated that the *Mab* genome shares considerable sequence similarity with that of *M. tuberculosis*. Consequently, the already generated data in TB drug discovery could also be used to identify new compounds active against *Mab* and other NTM [91]. Unfortunately, only a few antitubercular drugs, such as bedaquiline, are active against *Mab* growth, and are currently under evaluation for their use in therapy.

The *Mab* drug discovery is considered very challenging because of the typical lack of bactericidal activity, both in the currently recommended *Mab* treatment and in new tested compounds; it could be the reason for the poor therapeutic success in *Mab* infection [92]. For example, among the drugs currently used in *Mab* treatment, tigecycline and imipenem are bacteriostatic, while clarithromycin presents only a weak bactericidal activity at high concentrations [92]. The antitubercular bedaquiline is active against *Mab* growth, but it is also bacteriostatic (see below) [8]. The bacteriostatic activity could depend on the presence of several chromosomally encoded drug-modifying enzymes, the typical mycobacterial cell wall, or the presence of GLPs.

The new drugs that are active against *Mab* should, therefore, be bactericidal in order to be more effective. Moreover, new drug combinations, as well as repurposed compounds, are being tested against this pathogen.

**Figure 3:**
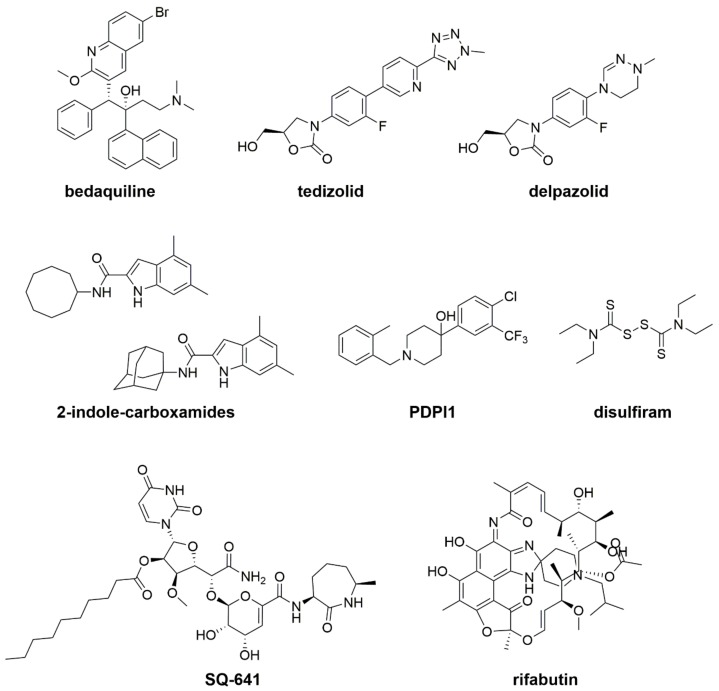
Anti-*Mab* drugs in preclinical and clinical studies.

Despite some active compounds being in preclinical development, only a few compounds are present in clinical trials (Table 2). It is noteworthy that several clinical trials include CF patients, highlighting the emerging role of *Mab* in this cohort of patients. Some drugs active against *Mab* growth, which are currently both in preclinical and in clinical trials, are described below (Table 2; Figure 3).

### 5.1. Bedaquiline

Bedaquiline (BED), an antitubercular drug belonging to diarylquinoline class, was approved by the U.S. Food and Drug Administration (FDA) in 2012 for the treatment of patients affected by pulmonary MDR- and XDR-TB only [118,119,120]. BED targets the mycobacterial ATP synthase (subunit c encoded by *atpE*) [120]. Another mechanism of resistance is linked to mutations in the *M. tuberculosis Rv0678* gene, which codes for the transcriptional repressor of the MmpS5-MmpL5 efflux system, causing cross-resistance between clofazimine and BED [90]. BED is also active against MAC and MABSC species, and its possible use is under evaluation because it lacks bactericidal activity, as previously described [93]. Moreover, *Mab* shares the same mechanism of BED resistance as *M. tuberculosis*; in fact, Mab_4383/Mab_4382 and Mab_4384 in *Mab* are homologous to MmpS5/MmpL5 and Rv0678 in *M. tuberculosis* [84,90]. Several BED-resistant *Mab* strains harbored mutations in the *Mab_4384* gene, and consequently an over-expression of MmpS5-MmpL5 efflux pump as a mechanism of resistance [84,94]. Recently, it was shown that verapamil, an efflux inhibitor, improved the BED activity against *Mab* clinical isolates, both in vitro and *ex vivo* [95]. 

Conflicting results were found when BED was tested in vivo, possibly because of its bacteriostatic activity; in a mouse model the BED treatment seemed to reduce the bacterial load [96], while in another murine model BED did not present any activity [97]. Interestingly, BED was highly effective in a zebrafish model of *Mab* infection; in particular, a short BED treatment was able to protect the *Mab*-infected larvae [98].

Overall, more studies are needed in order to understand if BED could be used against *Mab* infections, overtaking the problem related to its bacteriostatic activity and its mechanism of resistance. Different approaches could be performed, such as the use of BED in combination with an efflux inhibitor or the synthesis of new more effective BED derivatives.

### 5.2. New Oxazolidinone Derivatives: Tedizolid and Delpazolid

Linezolid, belonging to the oxazolidinone class, is administrated in NTM treatment, including *Mab* infections, but its clinical use is often related to adverse events. Recently, two other oxazolidinones were found to be more active against NTM than linezolid: tedizolid and delpazolid [99,100].

Currently, the most promising anti-*Mab* oxazolidinone is delpazolid, which is in phase II trials for TB treatment; it is active against *Mab* both in vitro and in vivo, likely without adverse effects [100].

### 5.3. MmpL3 Inhibitors: Indole-2-Carboxamides and PIPD1

Recently, MmpL3 inhibitors were found to be very effective against both *M. tuberculosis* and *Mab* [101,102,103,104,121].

Two lead compounds belonging to indole-2-carboxamides showed a potent bactericidal activity against *Mab* in vitro (MIC = 0.125 µg/mL) and *ex vivo*. Moreover, they were also active against a panel of *Mab* clinical isolates. It was shown that their cellular target is the mycolic acid transporter MmpL3. In particular, the indole-2-carboxamides strongly inhibited the transport of trehalose monomycolate, causing the loss of trehalose dimycolate production and abrogating mycolylation of arabinogalactan [101]. Moreover, these compounds showed minimal in vitro cytotoxicity and good selectivity indices [102]. Finally, in 2019 Pandya and collaborators tested the two lead compounds in vivo using a *Mab*- infected mouse model. Oral administration of the MmpL3 inhibitors showed a statistically significant reduction in bacterial load in the lungs and spleens of *Mab*-infected mice [103]. This last study confirms that indole-2-carboxamides are very promising anti-*Mab* compounds.

Dupont and collaborators (2016) performed a screening of a library of 177 anti-TB compounds against *Mab* growth. A new piperidinol-based molecule, PIPD1, was identified and characterized as having a potent bactericidal activity against *Mab*. Thanks to the isolation and characterization of some resistant *Mab* mutants, MmpL3 was identified as a cellular target [104]. Moreover, the treatment with PIPD1 in the *Mab*-infected zebrafish model decreased bacterial load and improved survival of the infected embryos [8].

MmpL3 represents the only new *Mab* drug target; moreover, the bactericidal activity of MmpL3 inhibitors indicates this target as one of the most druggable ones for this pathogen.

### 5.4. Capuramycin SQ641

Capuramycins are a novel class of nucleoside antibiotics targeting translocase-1, which is essential for peptidoglycan synthesis. SQ641 is the most active capuramycin against *Mab* growth (MIC = 0.25–1 µg/mL) and it is bactericidal. Furthermore, SQ641 showed synergy with rifabutin and streptomycin against *Mab* [105].

### 5.5. Repurposing and Repositioning Drugs: Rifabutin, Disulfiram, and β-Lactams

One of the strategies of discovery for new antimicrobial drugs is to reposition or to repurpose existing antibiotics, thus reducing the cost and time of their clinical development.

Following this aim, Aziz et al. (2017) identified rifabutin as a hit against *Mab* growth, starting from a screening of 2700 FDA-approved drugs [106]. Moreover, this drug is bactericidal against *Mab* and also effective *ex vivo*, even if other rifamycins are not active against this pathogen. Rifabutin has synergistic activity in several drug combinations, such as with amikacin, cefoxitin, linezolid, clarithromycin (in triple combination with tigecycline), and azithromycin [107].

Disulfiram is a drug used in the treatment of alcoholism. It was demonstrated that it has an antimicrobial activity against several microorganisms, and clinical trials for its use in treatment of a spectrum of diseases, such as HIV infection, are in progress. Surprisingly, disulfiram is active in vitro and *ex vivo* against *Mab*, showing time- and concentration-dependent killing, similar to amikacin [108]. It also synergized with moxifloxacin, ciprofloxacin, vancomycin, teicoplanin, and amikacin. In a murine model infected with *Mycobacterium fortuitum*, disulfiram significantly reduced bacterial counts in kidneys; consequently, it could also be active *in vivo* against *Mab* [108].

Currently, in the guidelines for treatment of *Mab* infections, only the two β-lactams cefoxitin and imipenem are included [109]. BlaMab is a very active β-lactamase encoded by a chromosomal gene [82], and it is responsible for the poor efficacy of β-lactams against *Mab*, as previously indicated. Interestingly, avibactam is a β-lactamase inhibitor, approved by the FDA in 2014, able to efficiently inhibit the β-lactamase BlaMab [110]. Recently, 206 paired combinations of antibiotics (β-lactams, β-lactamase inhibitors, rifamycins) were tested for in vitro synergy against *Mab* growth. Only 24 combinations exhibited synergy. Of these, 13 combinations included two β-lactams; 5 a β-lactam and avibactam; 6 included a β-lactam and a rifamycin. In the 5 combinations with avibactam, the MICs of three β-lactams (cefuroxime, imipenem, and biapenem) were reduced to below therapeutic breakpoints [111].

Pandey and collaborators (2019) discovered that the combinations of ceftazidime with either ceftaroline or imipenem were synergistic and had clinically relevant activities against clinical MABC isolates. Interestingly, these last β-lactams combinations were also active against THP-1 human macrophages infected with three different *Mab* clinical isolates [112].

Furthermore, the evaluation of these repositioning drugs (rifabutin and disulfiram, combinations of β-lactams) for their use in therapy against *Mab* is an interesting field of research and could complement the lack of new compounds against this pathogen. Even if more studies are needed, the recent findings of the inhibitory effect of avibactam against *Mab* β-lactamase support this research field.

### 5.6. Tigecycline

Tigecycline is a glycylcycline tetracycline in phase II of clinical development [83,113]. In this clinical trial, data were collected from 52 patients (58.3% with CF). Interestingly, in >60% of patients with *Mab* infections, including those with CF, an improvement was found when tigecycline was added to a multidrug treatment for ≥1 month [113,114].

### 5.7. Inhaled Formulation Nitric Oxide

An inhaled formulation of Nitric oxide (NO) is in phase II of clinical development for the treatment of *Mab* and the other NTMs [113].

NO is physiologically formed from L-arginine by NO synthase, and it plays an essential role in a variety of biological processes in the lung, including host defense against pathogens. Unfortunately, the airways of CF patients are NO-deficient, contributing to impaired lung function. Consequently, the increase of airway NO level is related to an improvement in lung function.

For this reason, NO is considered to be very promising against NTM infections. Bentur et al. (2019) evaluated the efficacy, safety, and tolerability of the intermittent inhaled NO in 9 CF patients with refractory *Mab* lung infection through a prospective, open labeled, multi-center, pilot study [115]. The treatment did not result in a *Mab* culture conversion (defined as 3 consecutive monthly negative culture of sputum samples), but caused a reduction in airway bacterial load. The main limitation of this study was the small number of CF-treated patients; additional clinical studies, for example with the use of larger cohorts of CF patients as well as an increase in the duration of NO therapy, are needed to understand the potential of this treatment [115].

### 5.8. Liposomal Amikacin for Inhalation

Liposomal amikacin for inhalation (LAI) is an inhaled drug in phase II of clinical development [113,116]. It is a novel formulation of amikacin, characterized by reduced toxicity and consequently an improved effectiveness in patients with refractory *Mab* lung disease. Caimmi and collaborators (2018) prescribed LAI to 5 CF patients with *Mab* infection at the Montpellier CF Center. Interestingly, 3 patients completed the treatment and did not have any respiratory exacerbation, showing negative cultures for *Mab* in their sputum [117].

Currently, another study is in progress, including 30 CF patients and the efficacy, safety, and tolerability of once-daily dosing of LAI 590 mg for 12 months [116].

### 5.9. Inhaled Molgramostim

Inhaled molgramostim is a formulation of granulocyte macrophage-colony-stimulating factor. It is a protein naturally occurring in the human immune system that plays an important role in activating the immune system to kill bacteria such as NTM. A phase 2 study is currently underway to test the effectiveness of inhaled mogramostim against NTM (including *Mab*) in adults with CF [113,116].

## 6. Conclusions and Future Perspectives

*Mab* is becoming one of the most frightening CF pathogens, as previously underlined. Clinical isolate sequencing has demonstrated that human-to-human *Mab* transmission is possible, further threatening CF patient health and contributing to its spread [14,43,44]. *Mab* virulence among CF patients is related to three important factors: (1) the transition from the S variant, important for colonization, to the R strain, fundamental for cell invasion [53]; (2) the GLPs of S strains, which are able to masque TDM and other lipids responsible for the activation of the innate immune system [51]; (3) the role of *CFTR* mutations in promoting *Mab* infections, as *CFTR* seems to have a specific role in the immune control of only this pathogen [69].

A key challenge for *Mab* infection treatment is to develop new strategies for its eradication, since, current therapy is poorly effective and no new drugs are on the horizon.

Alternative strategies, such as phage therapy, have already been investigated. Recently, after bilateral lung transplantation, a 15-year-old girl with CF and a disseminated *Mab* infection was successfully treated with a phage therapy [122,123]. This was the first therapeutic use of phages for a human mycobacterial infection, which was also well tolerated and without adverse reactions. This amazing result opens the possibility to use phage therapy against *Mab* infections in CF patients, even if accurate clinical trials are needed.

The finding of the β-lactamase inhibitor avibactam that effectively blocked Bla_Mab provides a new chance for the use of old antibiotics, such as β-lactams. Le Run and collaborators (2019) showed that the addition of avibactam improved the activity of the imipenem–tedizolid combination [124]. Interestingly, Lefebvre et al. (2017) demonstrated that the inhibition of Bla_Mab by avibactam improved the efficacy of imipenem against *Mab* *in vitro*, in macrophages and in zebrafish models [125], indicating that it should be clinically evaluated. It is noteworthy that the aztreonam–avibactam and ceftazidime–avibactam combinations are in phase I of clinical development for Gram-negative infections [113].

Among the possible new approaches, an interesting anti-virulence strategy could be exploited for inhibiting MAB_4780, the dehydratase required for cording formation [62], which is essential for *Mab* pathogenicity. This alternative approach to standard chemotherapy may represent an attractive way to attenuate cording, and consequently to control invasive and acute *Mab* infections, even among CF patients.

Another possible method for fighting drug-resistant *Mab* infections could be the design of Whib7 inhibitors that could be used in combination with the current therapy [79]. The inhibition of the activation of genes conferring aminoglycoside and macrolide resistance (*eis2* and *erm(41)*, respectively) in *Mab* could improve the efficacy of the current *Mab* treatment.

As described before, inhaled formulation or drugs or NO are also very promising in the fight against *Mab* infections. A further evaluation of efficacy and safety is needed prior to effective clinical use.

The emergence of *Mab* as a CF pathogen has left patients unprepared in the fight against this pathogen, which is intrinsically resistant to several classes of antibiotics already in use. Overall, a better understanding of *Mab* pathogenicity and more efforts in drug development pipeline could help to pave the way for next-generation antimicrobials that are effective in *Mab* treatment, in order to save more infected people.

## Figures and Tables

**Table 1:** Mechanisms of resistance to current drugs used against *Mab* infections.

Drugs	Targets	Mechanism of Resistance	Enzymes/Proteins Related to Mechanism of Resistance	References
**Macrolides**	23S rRNA	Mutations in target gene	Rrl (*MAB_r5052*)	[77,78]
		Modification of target	Erm(41) (*MAB_2297*)	[77]
		Induction of WhiB7 activator	Activation of *erm(41)* (*MAB_2297*)	[8,79]
**Amynoglicosides**	30S subunit of ribosome	Mutations in target genes	16S rRNA (*rrs*, *MAB_r5051*)	[80,81]
			RpsL (*MAB_3851c*)	
		Enzymatic drug modification	AAC(2′) (*MAB_4395*)	[75]
			Eis2 (*MAB_4532c*)	[75]
		Induction of WhiB7 activator	Activation of *eis2* (*MAB_4532c*)	[8,79]
**β-lactams**	Penicillin-binding protein	Enzymatic drug modification	Bla_Mab (*MAB_2875*)	[82]
**Tetracyclines**	30S subunit of ribosome	Enzymatic drug modification	MabTetX (*MAB_1496c*)	[83]
**Clofazimine**		Mutations in the repressor → Over-expression of an efflux pump	MAB_2299c	[84]
**Fluoroquinolones**	A subunit of DNA gyrase	Mutations in target gene	GyrA (*MAB_0019*)	[85,86]
		Other mechanisms?	not detected	[86]
**Linezolid**	23S rRNA	Mutations in target gene	Rrl (*MAB_r5052*)	[87]
		Efflux pumps?	LmrS and MmpL9?	

**Table 2:** Compounds in preclinical and clinical development against *Mab* infections.

Drugs	Development Phase	Target	Mechanism of Resistance	References
**Bedaquiline**	Preclinical studies	ATP synthase	MmpS5-MmpL5 efflux pump	[84,93,94,95,96,97,98]
**Tedizolid**	Preclinical studies	50S ribosome	-	[99]
**Delpazolid**	Preclinical studies	50S ribosome	-	[100]
**Indole-2-carboxamides**	Preclinical studies	MmpL3	-	[101,102,103]
**PIPD1**	Preclinical studies	MmpL3	-	[104]
**SQ641**	Preclinical studies	Translocase-1	-	[105]
**Rifabutin**	Preclinical studies	RNA polymerase		[106,107]
**Disulfiram**	Preclinical studies	-	-	[108]
**β-lactams (combinations)**	Preclinical studies	Penicillin-binding protein	Bla_Mab	[82,109,110,111,112]
**Tigecycline**	Phase II	30S subunit of ribosome	-	[83,113,114]
**Nitric oxide**	Phase II	-	-	[113,115,116]
**Liposomal Amikacin for Inhalation**	Phase II	23S rRNA	-	[113,116,117]
**Inhaled Molgramostim**	Phase II	-	-	[113,116]
